# Sex hormones regulate metainflammation in diet-induced obesity in mice

**DOI:** 10.1016/j.jbc.2021.101229

**Published:** 2021-09-29

**Authors:** Mita Varghese, Cameron Griffin, Simin Abrishami, Leila Eter, Nicholas Lanzetta, Layla Hak, Jeremy Clemente, Devyani Agarwal, Arianna Lerner, Maria Westerhoff, Ravi Patel, Emily Bowers, Mohammed Islam, Perla Subbaiah, Kanakadurga Singer

**Affiliations:** 1 Department of Pediatrics, Michigan Medicine, University of Michigan, Ann Arbor, Michigan, USA; 2 Department of Pathology, Michigan Medicine, University of Michigan, Ann Arbor, Michigan, USA; 3 Department of Mathematics and Statistics, Oakland University, Rochester, Michigan, USA

**Keywords:** obesity, macrophage, myelopoiesis, metabolism, sex differences, androgens, AR, androgen receptor, ATM, adipose tissue macrophage, BM, bone marrow, BMT, bone marrow transplantation, CAS, castration, CFU, colony-forming unit, CLS, crown-like structure, ER, estrogen receptor, ERα^−/−^, estrogen receptor–deficient alpha, G, granulocyte, GTT, glucose tolerance test, GWAT, gonadal white adipose tissue, GX, gonadectomy, HFD, high-fat diet, HSC, hematopoietic stem cell, ITT, insulin tolerance test, M, macrophage, ND, normal diet, OVX, ovariectomy, TG, triglyceride

## Abstract

Men have a statistically higher risk of metabolic and cardiovascular disease than premenopausal women, but the mechanisms mediating these differences are elusive. Chronic inflammation during obesity contributes to disease risk and is significantly more robust in males. Prior work demonstrated that compared with obese males, obese females have reduced proinflammatory adipose tissue macrophages (ATMs). Given the paucity of data on how sex hormones contribute to macrophage responses in obesity, we sought to understand the role of sex hormones in promoting obesity-induced myeloid inflammation. We used gonadectomy, estrogen receptor–deficient alpha chimeras, and androgen-insensitive mice to model sex hormone deficiency. These models were evaluated in diet-induced obesity conditions (high-fat diet [HFD]) and *in vitro* myeloid assays. We found that ovariectomy increased weight gain and adiposity. Ovariectomized females had increased ATMs and bone marrow myeloid colonies compared with sham-gonadectomized females. In addition, castrated males exposed to HFD had improved glucose tolerance, insulin sensitivity, and adiposity with fewer Ly6c^hi^ monocytes and bone marrow myeloid colonies compared with sham-gonadectomized males, although local adipose inflammation was enhanced. Similar findings were observed in androgen-insensitive mice; however, these mice had fewer CD11c^+^ ATMs, implying a developmental role for androgens in myelopoiesis and adipose inflammation. We concluded that gonadectomy results in convergence of metabolic and inflammatory responses to HFD between the sexes, and that myeloid estrogen receptor alpha contributes minimally to diet-induced inflammatory responses, whereas loss of androgen-receptor signaling improves metabolic and inflammatory outcomes. These studies demonstrate that sex hormones play a critical role in sex differences in obesity, metabolic dysfunction, and myeloid inflammation.

Men and women of reproductive age respond differently to obesity, with men exhibiting higher rates of diabetes and cardiovascular disease than women despite similar obesity rates (1). Premenopausal women are less prone to developing metabolic disease than age-matched or body mass index–matched men (2), whereas postmenopausal women develop increased risk of obesity-related disorders, suggesting a potential role for sex hormones in these discrepancies (3). Similarly, hypogonadal men, regardless of body mass index, have an increased risk for metabolic disease (4). The increase in cardiometabolic risk after menopause and in hypogonadal men is paralleled by the redistribution of body fat and is reversed or decreased by sex hormone replacement therapy (5, 6, 7). Visceral adiposity is strongly associated with metabolic syndrome (8). When fed a high-fat diet (HFD), male mice gain significantly more adipose mass and exhibit profound metabolic dysfunction compared with female mice fed HFD (9). While HFD-fed female mice exhibit an increase adipocyte size, they remain metabolically protected from the negative consequences of obesity, such as hyperglycemia and insulin resistance (9, 10).

The mechanisms behind this sexual dimorphism in adipose dysfunction remain unclear. However, there is strong evidence to implicate a shift in immune cell responses to a more chronic activated myeloid profile that contributes to sequelae of obesity in males (11, 12). Specifically, proinflammatory CD11c^+^ adipose tissue macrophages (ATMs) predominate in males, whereas CD11c^−^ ATMs increase in females (13). This immune activation in adipose tissue is driven by adipocyte expansion, adipocyte death, and lipolysis (14, 15). Adipogenesis has been demonstrated to be sexually dimorphic (16). We have previously demonstrated that with short-term HFD adipose expansion and induced lipolysis, both males and females have robust proliferation of CD11c^−^ ATMs (17, 18). However, we have also seen that in obese males, hematopoietic stem cells (HSCs) and myeloid progenitors expand with dietary lipid exposure, and recruitment signals within tissues drive a sustained inflammatory tone with the accumulation of monocytes and CD11c^+^ ATMs (17, 19).

A variety of models have been used to understand the role of sex hormones in metabolism and adipose tissue health. Estrogens signaling through their cognate estrogen receptors (ERs) play a protective role in glucose metabolism, food intake, and peripheral insulin sensitivity (10, 20). Androgens also play multifaceted roles in insulin production, body composition, and metabolism (21). Mice with aromatase deficiency, which blocks the conversion of testosterone to estrogen, have increased visceral adiposity and liver steatosis (22) emphasizing the importance of estrogen synthesis in regulating lipid storage. Androgens also directly impact metabolism *via* activation of the androgen receptor (AR) on adipocytes, which has been found to regulate adipogenesis (23, 24). Overall, while these studies indicate that sex hormones and their intermediates have a sexually dimorphic role in metabolism, they have not directly compared sexually dimorphic inflammatory responses or delineated the importance of specific sex hormone receptors to inflammation phenotypes.

Our prior work demonstrated that males and females on HFD have weight gain, adipocyte hypertrophy, and differential macrophage polarization. Males expand myeloid progenitors in the bone marrow (BM) leading to increased circulating monocytes and increased tissue myeloid cells (13, 25), contributing to insulin resistance, whereas females are protected from this metabolic inflammation. Although the difference in inflammatory responses is well established and strongly implicated in the discrepancies observed in disease manifestation between the sexes, little is known about the catalyst for this dimorphism. We hypothesize that sex hormones modify obesity-induced disease manifestations in males and females and contribute to the observed inflammatory shift.

To further investigate the relationship between sex hormones and tissue myeloid inflammatory responses in obesity, we conducted studies using the following mouse models of sex hormone deficiency: (1) gonadectomy (GX); (2) mice lacking signaling in the AR (AR^tfm^); or (3) ER-deficient alpha (ERα^−/−^). Our studies demonstrate that GX results in convergence of metabolic inflammatory responses in male and female mice. Contrary to prior reports that estrogen dampens inflammatory responses (26, 27, 28), we found limited effects of hematopoietic ERα deficiency in metabolism or diet-induced inflammation. In contrast, AR deficiency resulted in significant improvement in metabolic and inflammatory responses to HFD.

These studies demonstrate the complexity of GX and that the role of sex hormones in diet-induced obesity cannot be attributed to single hormone/receptor interactions.

## Results

### GX attenuates sex differences in metabolic responses to obesity

Our previous studies with WT mice challenged with 60% HFD for 16 weeks showed that glucose intolerance, insulin resistance, and adipose tissue inflammation develop primarily in male mice, whereas female mice remained protected (13). Earlier studies with gonadectomized mice demonstrated differential susceptibility to obesity between male, female, and ovariectomized female mice but did not address inflammatory responses (29). Critically, prior studies have compared intact males with only gonadectomized females and thus neglected the effect of androgens in diet-induced obesity (29). Prior studies that only compared intact males to gonadectomized females and involved diets that varied in fat percentages and duration failed to delineate the effect of androgens in diet-induced obesity (30, 31, 32). A direct comparison of gonadectomized males and females to gonadally intact animals is also necessary to determine the specific role of androgens and estrogens on metabolic and inflammatory responses.

**Figure 1:**
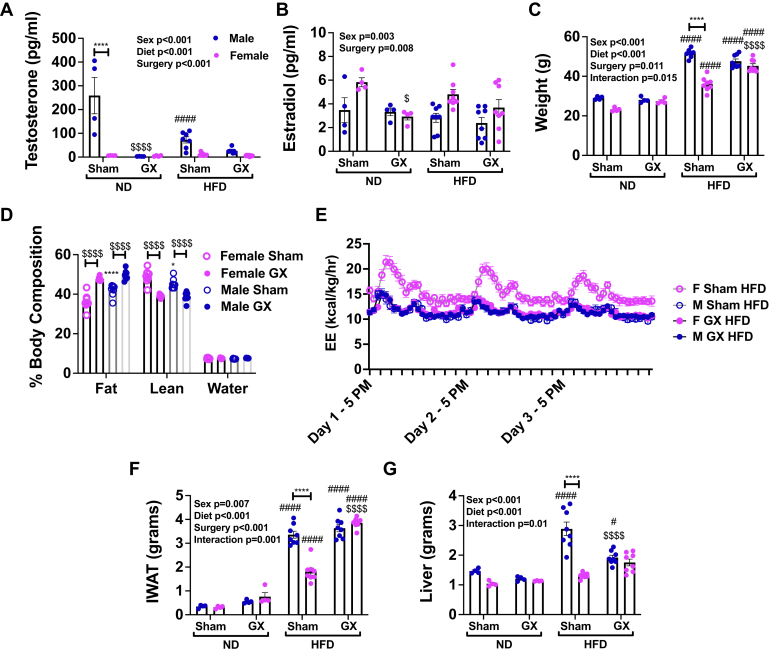
**Gonadectomy (GX) reduces sex hormones and increases adiposity.** Male and female animals underwent sham surgery or GX at 4 weeks of age and then at 6 weeks of age remained on ND or started HFD. Serum testosterone (*A*) varied significantly by sex, surgery, and diet, with significant interaction among all factors (*p* < 0.001 for all) and was lowered by GX in male ND animals ($$$$) and by HFD in sham male animals (####). Serum estradiol (*B*) varied significantly by surgery and sex and was lowered by GX in female ND animals. Body weight (*C*) varied significantly by sex, surgery, and diet with significant interaction among all factors. Female sex showed attenuated weight gain in sham animals, whereas GX HFD animals of both sexes gained equal weights. Body composition (*D*) demonstrated an increase in fat mass with GX in both sexes and an increase in fat mass in male sham compared with female sham. *E*, energy expenditure (EE) was highest in female sham HFD mice. IWAT (*F*) and liver (*G*) weight showed a greater increase in male than female sham HFD animals. With GX, there was no difference by sex in these tissue weights. N = 4 for ND groups and N = 8 for HFD groups. Data shown as average ± SEM. ∗*p* < 0.05, ∗∗*p* < 0.01, ∗∗∗*p* < 0.005, and ∗∗∗∗*p* < 0.001 between male and female of each group. ^#^*p* < 0.05, ^##^*p* < 0.01, ^###^*p* < 0.005, and ^####^*p* < 0.001 marks differences between ND and HFD of the same sex and surgery group. ^$^*p* < 0.05, ^$$^*p* < 0.01, ^$$$^*p* < 0.005, and ^$$$$^*p* < 0.001 marks significant differences by surgery. HFD, high-fat diet; IWAT, inguinal white adipose tissue; ND, normal diet.

To address this, we conducted studies in hormone-deficient mouse models. We performed sham or GX procedures at 4 weeks of age in males (castration [CAS]) and females (ovariectomy [OVX]) and then challenged them with 60% HFD at 6 weeks of age. To understand the effects of GX on sex hormones, we evaluated testosterone and estradiol levels in serum of male and female GX mice. As expected, a marked reduction in testosterone was observed with CAS in males (Fig. 1*A*). HFD alone also reduced testosterone levels in males. Testosterone levels were similar in GX normal diet (ND) and GX HFD male animals. Overall, estradiol was lowered by GX in females (Fig. 1*B*). However, the effect size of GX and male/female differences in estradiol levels were moderate compared with the effect of GX on testosterone.

Body weight comparisons showed that all HFD-fed groups gained weight compared with their ND controls (Fig. 1*C*). While GX animals of both sexes had similar body weights in their respective diet conditions (Fig. 1*C*), they manifested increased fat mass compared with their gonadally intact sham counterparts (Fig. 1*D*). Overall, female sham HFD mice had the highest energy expenditure (Fig. 1*E*) and lowest body weight gain. Consistent with body weight equalization between sexes with GX, inguinal white adipose tissue and liver mass were also similar in GX HFD males and females (Fig. 1, *F* and *G*), because of an increase in inguinal white adipose tissue in female GX HFD animals and a decrease in liver mass in male GX HFD animals. Overall, the effect of GX is to markedly reduce testosterone in males and moderately reduce estradiol in females, which is associated with convergence of weight, energy expenditure, and body composition among males and females in response to HFD.

**Figure 2:**
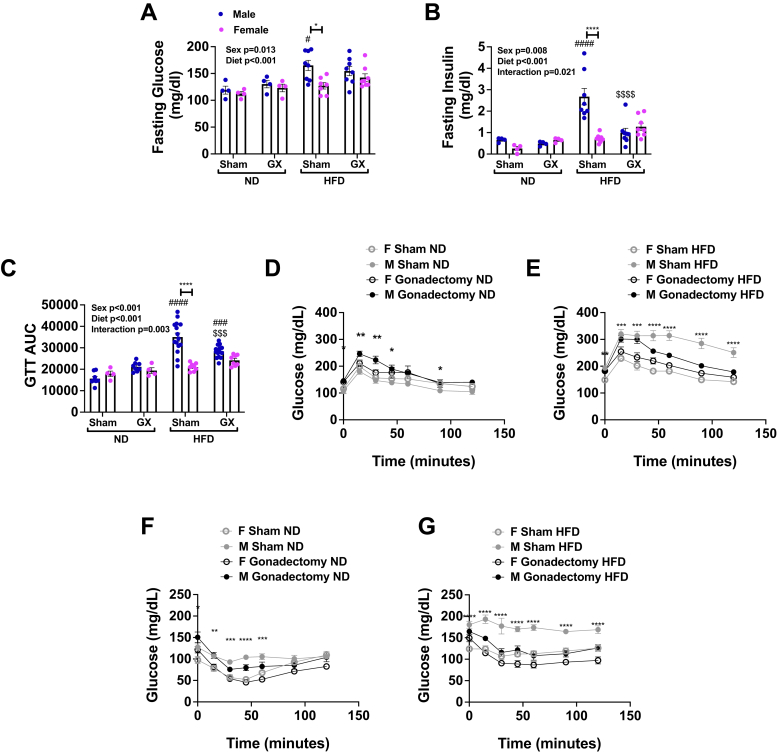
**Gonadectomy (GX) leads to improved metabolism in male HFD-fed animals.** Male and female animals underwent sham surgery or GX at 4 weeks of age and then at 6 weeks of age remained on ND or started HFD. After 10 weeks fasting, glucose (*A*) varied significantly by sex and diet driven by differences in the sham macrophage (M) HFD group. Fasting insulin (*B*) was highest in the sham M HFD group and was significantly decreased by GX. This pattern was also observed in 12-week GTT data as area under the curve (AUC) (*C*) and GTT time courses (*D*, ND and *E*, HFD). Insulin tolerance tests showed that insulin sensitivity was lowest in M sham ND and HFD mice (*F*, ND and *G*, HFD). Data shown as average ± SEM. ∗*p* < 0.05, ∗∗*p* < 0.01, ∗∗∗*p* < 0.005, and ∗∗∗∗*p* < 0.001 between male and female of each group. ^#^*p* < 0.05, ^##^*p* < 0.01, ^###^*p* < 0.005, and ^####^*p* < 0.001 marks differences between ND and HFD of the same sex and surgery group. ^$^*p* < 0.05, ^$$^*p* < 0.01, ^$$$^*p* < 0.005, and ^$$$$^*p* < 0.001 marks significant differences by surgery. GTT, glucose tolerance test; HFD, high-fat diet; ND, normal diet.

In gonadally intact male mice, within 10 weeks of HFD, fasting glucose and insulin levels were greatest in male HFD sham compared with female HFD sham and male ND (Fig. 2*A*). However, GX decreased insulin levels in male GX HFD animals to levels comparable to female sham and GX HFD groups (Fig. 2*B*). Glucose tolerance tests (GTTs) in ND-fed mice demonstrated that GX in male mice worsened glucose intolerance, with higher glucose area under the curve (Fig. 2*C*) and peak glucose levels (Fig. 2*D*) in male GX animals, when compared with male sham animals and both sham and GX females. However, this effect was reversed in HFD-fed animals; at 12 weeks of HFD, male GX HFD animals had improved glucose tolerance compared with male sham HFD animals (Fig. 2*E*). Insulin tolerance tests (ITTs) conducted in the same animals revealed no main effect of GX in ND although there was a sex effect with both sham and GX females having lower glucose values than males (Fig. 2*F*). In contrast, in the setting of HFD, male sham HFD animals had the highest glucose levels, and GX resulted in a significant improvement in insulin sensitivity in male GX HFD animals (Fig. 2*G*). Levels in GX male HFD mice were comparable to that seen in female GX HFD and female sham HFD groups.

After 16 weeks, fed glucose levels were significantly altered by diet and GX. Female GX HFD animals had significantly higher fed glucose levels compared with female sham HFD mice, with male sham HFD animals having the highest insulin levels overall (Fig. S1, *A* and *B*). Further evaluation of gonadal white adipose tissue (GWAT) demonstrated that *Ir* and *Irs1* (representing insulin receptor response) gene expression was higher in female sham HFD compared with male sham HFD animal (Fig. S1, *D* and *E*), GX in HFD females lowered expression, whereas in male HFD, it increased gene expression of these insulin-signaling genes. Consistent with this, male sham HFD mice had higher fasting homeostatic model assessment for insulin resistance compared with female sham HFD mice, and this was improved in male GX HFD mice, suggesting increased insulin sensitivity in GX males (Fig. S1*F*). No significant changes in homeostatic model assessment for insulin resistance were seen in GX females. These metabolic studies show that in sham animals, males had more metabolic consequences of HFD than females, and GX attenuated those differences.

### GX dampens sex differences in meta-inflammation

**Figure 3:**
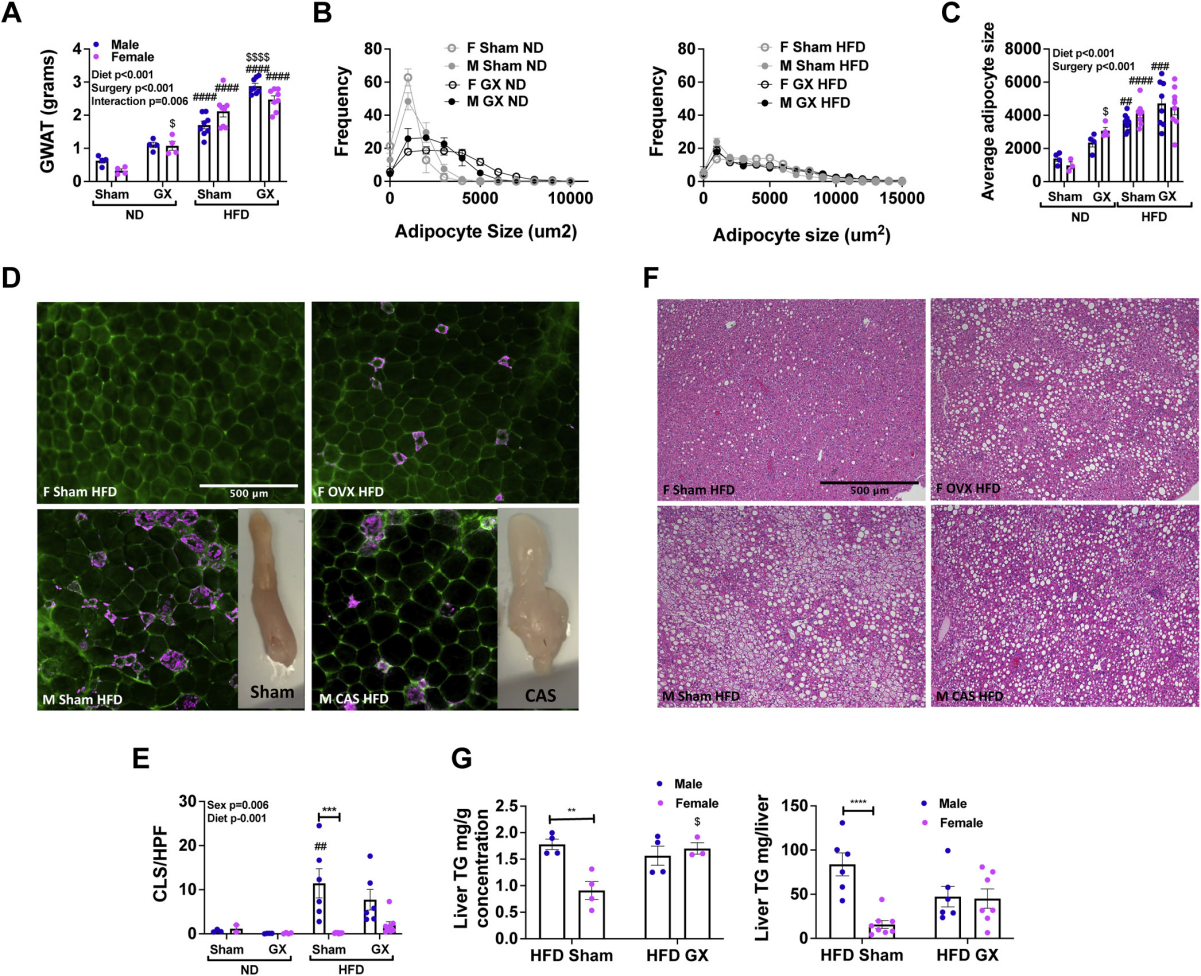
**Gonadectomy (GX) alters adipose tissue and liver lipid storage in HFD.** Male and female animals underwent sham surgery or GX at 4 weeks of age and then at 6 weeks of age remained on ND or started HFD. Visceral fat was evaluated as GWAT mass (*A*), which varied significantly by diet and surgery, with highest overall values in GX animals of both sexes. GWAT adipocyte size was evaluated by distribution (*B*) and average adipocyte size (*C*). Concordant with mass, adipocyte size was greatest in GX HFD of both sexes. Immunofluorescence of GWAT (caveolin, *green* and Mac2 staining, *magenta*) (*D*) and GWAT crown-like structures/high-power field (*E*) varied by sex and diet and was greatest in male HFD animals with no significant effect of GX. *F*, liver H&E staining. *G*, in HFD-exposed animals, liver triglyceride (TG) estimation per gram of liver and total liver mass was performed. Male mice have higher liver TG than female mice, an effect that was attenuated by increased liver TG in females after GX. *H*, liver H&E sections scored for nonalcoholic steatohepatitis (NASH). N = 4 to 8 per group. Data shown as average ± SEM. ∗*p* < 0.05, ∗∗*p* < 0.01, ∗∗∗*p* < 0.005, and ∗∗∗∗*p* < 0.001 between male and female of each group. ^#^*p* < 0.05, ^##^*p* < 0.01, ^###^*p* < 0.005, and ^####^*p* < 0.001 marks differences between ND and HFD of the same sex and surgery group. ^$^*p* < 0.05, ^$$^*p* < 0.01, ^$$$^*p* < 0.005, and ^$$$$^*p* < 0.001 marks significant differences by surgery. GWAT, gonadal white adipose tissue; HFD, high-fat diet; ND, normal diet.

Prior studies have demonstrated that in males, GX leads to adipocyte hypertrophy, increased lipogenesis, and increased inflammatory gene expression (33). However, it is not known if loss of sex hormones also alters adipose tissue and inflammation in females. After 16 weeks of HFD challenge, GX animals had increased total mass of visceral GWAT regardless of sex (Fig. 3*A*). This equalized between sexes when GWAT mass was normalized to body weight (Fig. S1*C*). In ND animals, there were no sex differences in average adipocyte size, but GX increased average adipocyte size in males and females (Fig. 3, *B* and *C*), whereas in HFD, there was no effect of GX (Fig. 3, *B* and *C*).

To investigate the role of sex hormones in inflammatory responses, we performed tissue-specific histology and flow cytometry studies. Crown-like structure (CLS; macrophages accumulated around adipocytes) density was increased most markedly in male gonad-intact HFD mice relative to corresponding females (Fig. 3, *D* and *E*). Of note, there is no significant difference in CLS density among male and female HFD mice after GX. The moderate increase in CLS density in female HFD GX animals compared with its gonad-intact counterpart—although it did not reach significance—combined with marked increase in GWAT mass (Fig. 3*A*) raises the question of whether overall burden of adipose inflammation is increased in those animals.

Male sham HFD animals had micro (small cellular lipid droplets) and macrosteatosis (large lipid droplets), but both female and male GX HFD animals had only macrosteatosis (Fig. 3*F*) and similar liver triglyceride (TG) content (Fig. 3*G*). Possibly as a consequence of changes in adiposity, liver histology showed the presence of liver steatosis in female GX HFD mice at levels similar to male GX HFD (Fig. 3, *E*–*G*). These results demonstrate that GX leads to convergence in adiposity and liver steatosis between sexes.

**Figure 4:**
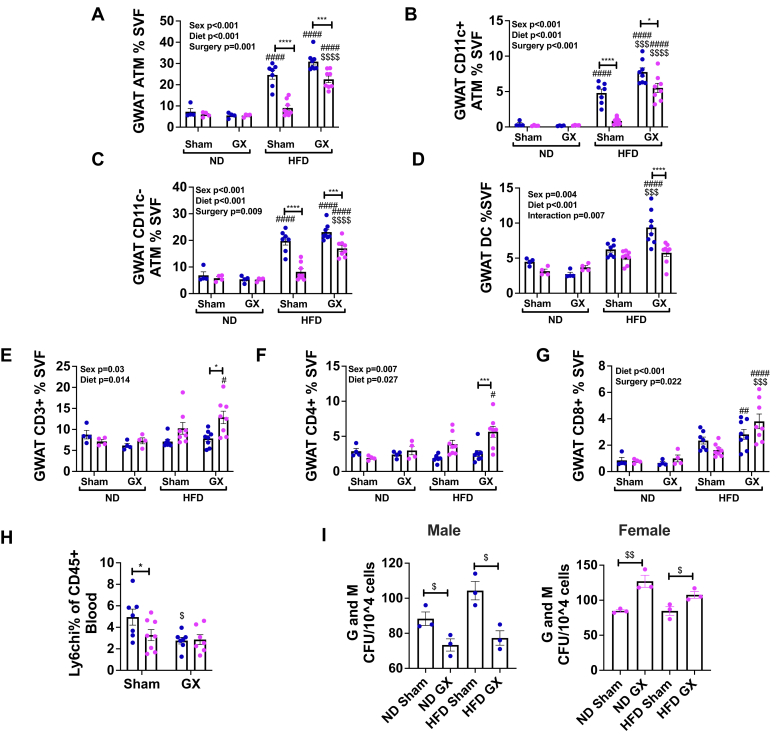
**Gonadectomy (GX) alters adipose tissue leukocytes and BM myeloid responses to HFD.** Male and female animals underwent sham surgery or GX at 4 weeks of age and then at 6 weeks of age remained on ND or started HFD. Flow cytometry was conducted on the visceral fat (GWAT) stromal vascular fraction (SVF). *A*, as percent of SVF adipose tissue macrophages (ATMs) varied significantly by sex, diet, and surgery and overall highest in male HFD animals regardless of GX. GX markedly increased ATMs in female HFD animals. The same pattern was seen in CD11c^+^ ATMs (*B*) and CD11c^−^ ATMs (*C*). Dentritic cells (DCs) (*D*) however were increased in GX males. *E*–*G*, CD3^+^ T cells as well as the CD4^+^ and CD8^+^ subsets varied significantly only in female GX animals. Circulating Ly6c^hi^ monocytes (*H*) were highest in male sham HFD mice. To next understand the influence on BM myelopoiesis, myeloid methylcellulose colony-forming units (CFUs) were counted. *I*, granulocyte (G) and macrophage (M) were assessed. Male GX mice had a reduction in myeloid colonies, whereas female GX mice had an increase. N = 3 for CFU assay. N = 4 to 8 per group. Data shown as average ± SEM. ∗*p* < 0.05, ∗∗*p* < 0.01, ∗∗∗*p* < 0.005, and ∗∗∗∗*p* < 0.001 between male and female of each group in panels *A*–*G*. ^#^*p* < 0.05, ^##^*p* < 0.01, ^###^*p* < 0.005, and ^####^*p* < 0.001 marks differences between ND and HFD of the same sex and surgery group. ^$^*p* < 0.05, ^$$^*p* < 0.01, ^$$$^*p* < 0.005, and ^$$$$^*p* < 0.001 marks significant differences by surgery. BM, bone marrow; GWAT, gonadal white adipose tissue; HFD, high-fat diet; ND, normal diet.

Given the difficulties in quantifying CLSs in heterogeneous adipose tissue by histology, flow cytometric analysis of GWAT immune cells was conducted. In visceral adipose tissue GWAT, ATMs were increased in male sham HFD compared with male ND and female sham HFD. However, ovariectomy increased GWAT ATMs in female HFD mice compared with ovary-intact mice (Fig. 4*A*). In HFD females, GX increased ATMs of the CD11c^+^ and CD11c^−^ type (Fig. 4, *B* and *C*). In male mice, GX did not change the overall prevalence of ATMs but did increase CD11c^+^ ATMs (Fig. 4*B*), consistent with prior reports (33). Dendritic cell numbers were highest in male GX HFD animals and remained unchanged in females after GX (Fig. 4*D*). Adipose tissue T-cells of both the CD4 and CD8 types were higher in GX HFD females (Fig. 4, *E*–*G*). Gene expression studies to further assess inflammatory markers in adipose tissue showed increased proinflammatory *Il6* and *Mcp1* expression in GX animals of both sexes in response to HFD (Fig. S1, *G* and *H*). Regulatory *Il4* had higher expression in all female groups (Fig. S1*I*). In summary, GX worsened adipose tissue inflammation in female mice exposed to HFD but surprisingly did not improve adipose tissue inflammation in male mice despite improvement in glucose tolerance and hepatic TG content.

Given the effects of GX on CD11c^+^ ATMs, which are typically recruited from circulation, we next evaluated Ly6c^hi^ monocytes in circulation and proliferation of BM progenitors. Surprisingly, male HFD sham animals had the highest percentage of Ly6c^hi^ monocytes in circulation, whereas GX male mice showed reduced numbers (Fig. 4*H*), despite the increase in CD11c^+^ ATM with GX. Given that a major source of recruited ATMs in chronic HFD is from the BM, we assessed the effect of GX on *in vitro* proliferation of myeloid colonies in GX male mice. Myeloid colonies were counted as colony-forming units (CFUs). This *in vitro* assessment of BM myeloid potential evaluates the BM propensity to generate macrophages and neutrophils. When CFUs were further classified as granulocytes (G) and macrophages (M), we identified that in HFD conditions, GX decreased the production of myeloid G and M colonies in males but increased them in females in both ND and HFD conditions (Fig. 4*I*). These results in the BM are discordant with the abundance of CD11c^+^ ATM in tissue, which were increased by GX in HFD animals. This discordance suggests that GX may play a significant role in altering the tissue microenvironment, rather than exerting an effect only through BM myeloid cell production.

Overall, our findings demonstrate that GX results in convergence of metabolic phenotypes in males and females when exposed to HFD. Worsening adipose tissue inflammation in GX females was associated with marked changes in energy expenditure but only minor changes in glucose metabolism. In males, GX significantly worsened metabolic outcomes with minor changes to adipose tissue inflammation. While endocrine manipulation clearly changed metabolic outcomes, the complex changes in phenotypes observed after GX bring up the question of whether non-gonadal sources of sex hormones significantly effect immune responses to HFD in ways not reflected in circulating hormone levels. We therefore proceeded to examine the necessity of ER and testosterone receptor signaling for immune responses to HFD.

### Hematopoietic ERα^−/−^ deficiency does not change metainflammation but augments BM myeloid expansion *in vitro*

Given that HFD GX females had an enhanced inflammatory tone compared with HFD sham females, we sought to next understand the effects of estrogens on driving metainflammation in males and females. To investigate this, we generated animals that were hematopoietically deficient in ERα *via* BM chimeras. ERα is known to be the predominant ER in myeloid cells and adipose tissue (26, 28). We thus used BM chimeras given that whole-body ERα^−/−^ mice have bone fragility and can be unsuitable for long-term experiments especially in the context of weight gain (26, 34). Comparisons were performed between ERα^−/−^ → WT (ERα^−/−^ BM transplanted into WT) and WT → WT (WT BM transplanted into WT) for both male and female mice. After BM reconstitution and recovery, a subgroup of animals was challenged to HFD.

**Figure 5:**
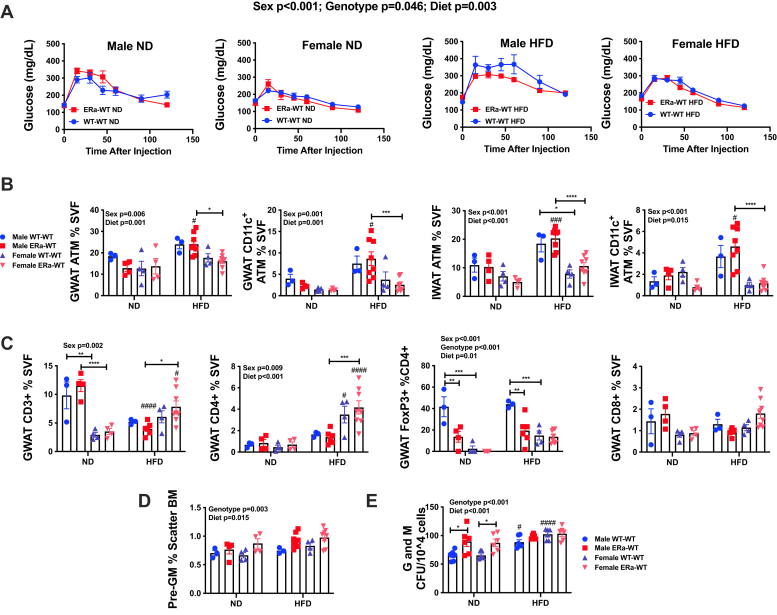
**Hematopoietic ERα**^**−/−**^**does not influence metabolism and BM responses to HFD.** Male and female bone marrow transplants (BMTs) were generated with either ERα^−/−^ or WT BM. Animals were placed on either ND or HFD. Donor and recipient sex were the same in these studies. *A*, GTT was performed at 10 weeks of diet. While repeated-measures analysis of glucose responses revealed a weak effect of genotype, overall when diet and sex conditions were tested, there was no significant pairwise difference because of ERα^−/−^. Flow cytometry of GWAT and IWAT ATMs (*B*) likewise showed significant differences by sex and diet but no influence of ERα^−/−^. GWAT adipose tissue T-cells (*C*) only demonstrated a difference in genotype in FoxP3^+^ T cells in males. Pre–granulocyte and macrophage (pre-GM) progenitors in bone marrow (*D*) significantly varied by diet and genotype. Consistent with this, BM myeloid methylcellulose colony-forming units (CFUs) (*E*) showed increased G and M myeloid colonies in ERα^−/−^ BM of both sexes; however, this was not significantly different in animals challenged to HFD. N = 4 to 8 per group. Data shown as average ± SEM. ∗*p* < 0.05, ∗∗*p* < 0.01, ∗∗∗*p* < 0.005, and ∗∗∗∗*p* < 0.001. ^#^*p* < 0.05, ^##^*p* < 0.01, ^###^*p* < 0.005, and ^####^*p* < 0.001 marks differences between ND and HFD of the same sex and donor group. ATM, adipose tissue macrophage; BM, bone marrow; ERα^−/−^, ER-deficient alpha; GTT, glucose tolerance test; GWAT, gonadal white adipose tissue; HFD, high-fat diet; IWAT, inguinal white adipose tissue; ND, normal diet.

After 10 weeks of diet challenge, fasting glucose levels were similar in all groups (Fig. S2*A*). GTT showed changes in glucose tolerance related to sex and diet (Figs. 5*A* and S2*B*) concordant with those observed in GX experiments (Fig. 2), but no changes were observed because of ERα^−/−^ donor genotype. There were no significant differences in body or GWAT weights based on donor genotype (Fig. S2*C*). Likewise, ERα^−/−^ genotype did not significantly alter ATMs (Fig. 5*B*) or T cells (Fig. 5*C*), with the exception of FoxP3^+^ CD4 cells, which were decreased in males transplanted with BM from ERα hematopoietic knockouts in both ND and HFD conditions.

Given that we did not observe a significant effect of hematopoietic ERα deficiency *in vivo*, we sought to verify previously reported effects of ERα (26, 28). We evaluated BM progenitors by flow cytometry and myeloid colony assays *in vitro*. Consistent with prior studies showing that ERα influences inflammatory responses, WT groups transplanted with BM from hematopoietic knockout animals had significant differences compared with WT animals in BM pre-GM myeloid progenitors and BM-derived myeloid colonies (Fig. 5, *D* and *E*) with an increase in progenitors in ERα^−/−^ marrow across diet and sex. ERα^−/−^ genotype did not recapitulate the increase in BM CFUs observed in HFD GX females. Post hoc analysis demonstrated that ND male ERα^−/−^ → WT generated more myeloid CFUs compared with male ND WT → WT animals (Fig. 5*E*), but after HFD, all groups were similar in myeloid CFU response. Interestingly, early HSC progenitors, multipotent progenitors, and hematopoietic progenitor cell 1 did not appear to be different, but late progenitors such as hematopoietic progenitor cell 2 were decreased in all ERα^−/ −^ → WT groups (Fig. S2). This decrease in less committed progenitors and increase in more myeloid committed progenitors suggests that estrogen may influence skewing or differentiation of later progenitors (35). Overall, the results of hematopoietic ERα^−/−^ fail to recapitulate the moderate changes seen in adipose tissue inflammation seen in female mice after GX. Given that there is not a large effect of OVX on estradiol levels, this could simply reflect that changes in estrogen alone are not the primary source of metabolic and inflammatory changes after OVX in HFD.

### Developmental androgen signaling plays a role in obesity-induced adipose tissue and myeloid inflammation

**Figure 6:**
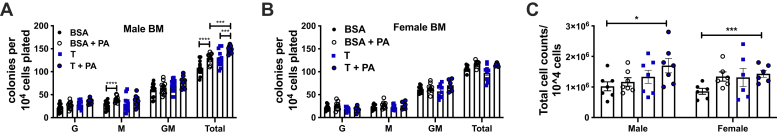
**Male and female BM has exaggerated *in vitro* myeloid stimulation with palmitate after testosterone exposure.** To understand the impact of palmitate and/or testosterone, myeloid methylcellulose CFU assays were plated from WT male (*A*) and female mice (*B*). There was a stepwise increase in myeloid colonies in males with the addition of palmitate and testosterone. Total cell number counts after 7 days (*C*) did show a significantly higher number of cells in male and female plates treated with a combination of testosterone and palmitate. N = 9 to 15 for CFU assays. Data shown as average ± SEM. ∗*p* < 0.05, ∗∗*p* < 0.01, ∗∗∗*p* < 0.005, and ∗∗∗∗*p* < 0.001. BM, bone marrow; CFU, colony-forming unit.

Because of the profound effect of GX on testosterone levels in male mice, we examined the role of AR signaling in myeloid cell proliferation and metabolic and inflammatory responses to HFD. We performed *in vitro* methylcellulose assays with BM cells. We tested *in vitro* addition of testosterone in the presence of a dietary lipid—palmitate—to determine whether this treatment shifted the predisposition of the BM from ND WT male and female mice. This addition of the saturated fatty acid, palmitate is a tissue culture model used to mimic the response to diets high in saturated fat (17). Consistent with the decrease in BM myeloid responses with androgen loss through castration (Fig. 4*H*), the addition of testosterone in ND male BM cells enhanced the number of total granulocyte (G) and macrophage (M) colonies in males (Fig. 6*A*), whereas there were no significant changes in females (Fig. 6*B*). However, testosterone in the presence of palmitate increased the total cell numbers harvested from plates in both males and females (Fig. 6*C*). This indicates that combined palmitate and testosterone treatment expands the progenitor pool in males only but increases proliferation rates in both males and females.

**Figure 7:**
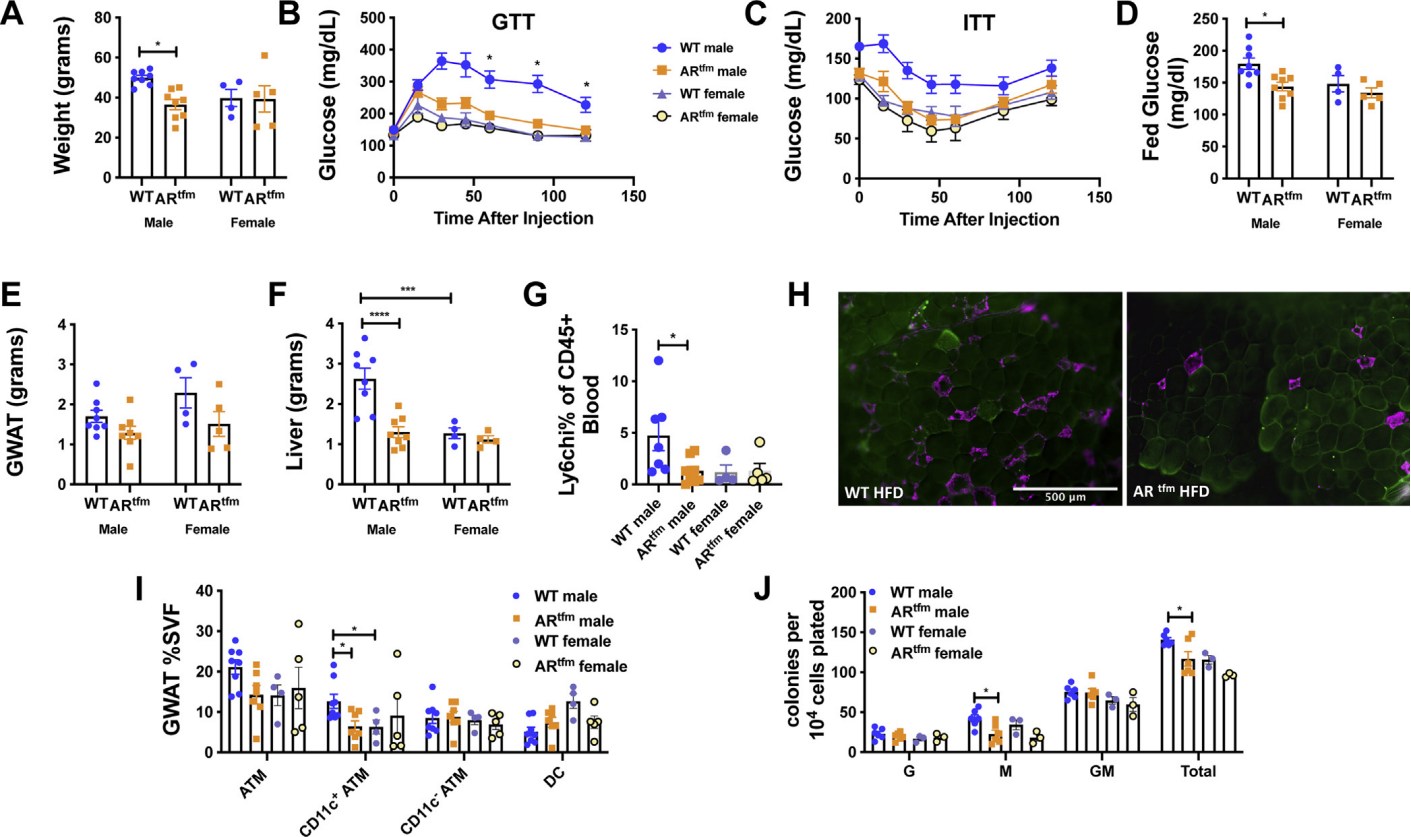
**Male AR**^**tfm**^**animals on HFD have attenuated metainflammation.** WT and AR^tfm^ male and female mice were placed on ND or HFD starting at 6 weeks of age. Weight gain (*A*), GTT (*B*), ITT (*C*), and fed glucose levels (*D*) were all significantly worsened in male WT mice and improved to the level of female animals by AR deficiency. There was no effect of AR^tfm^ in female mice. GWAT weights (*E*) were significantly different by genotype (*p* = 0.017), but there was no post hoc difference in males or females. Liver weights (*F*) were highest in WT males and normalized by AR^tfm^. Both circulating Ly6c^hi^ monocytes (*G*) and CD11c^+^ ATMs (*H*, immunofluorescence [caveolin, *green* and Mac 2, *magenta*], *I*) were highest in WT males. *J*, AR^tfm^ reduced myeloid and total granulocyte and macrophage colonies in HFD males. N = 4 to 8 per group of mice. Data shown as average ± SEM. ∗*p* < 0.05, ∗∗*p* < 0.01, ∗∗∗*p* < 0.005, and ∗∗∗∗*p* < 0.001. AR, androgen receptor; GTT, glucose tolerance test; GWAT, gonadal white adipose tissue; HFD, high-fat diet; ITT, insulin tolerance test; ND, normal diet.

To further understand the role of androgens, we conducted studies using the AR^tfm^ (testicular feminization) mouse as a model of AR deficiency, given lack of androgen signaling responses in this animal from the time of development as opposed to postnatal GX. As in the previous experiments, animals were challenged with HFD for 16 weeks. Male AR^tfm^ animals were of lower weight compared with WT males (Fig. 7*A*), had improved glucose tolerance, insulin sensitivity (Fig. 7, *B* and *C*), and lower-fed glucose than WT males (Fig. 7*D*). While there were no significant differences in visceral GWAT (Fig. 7*E*), male AR^tfm^ mice were protected from liver expansion as predicted from GX studies (Fig. 7*F*). On the other hand, female AR^tfm^ mice did not show any significant changes in liver mass.

Consistent with male sex hormones driving metainflammation, male AR^tfm^ mice fed HFD had fewer Ly6c^hi^ monocytes in blood (Fig. 7*G*) and fewer GWAT CLSs (Fig. 7*H*) than WT males and females. Flow cytometry showed fewer CD11c^+^ ATMs in GWAT (Fig. 7*I*) compared with WT males and females. To next determine if androgen depletion affects hematopoietic myeloid progenitors, *in vitro* myeloid expansion assays were performed. BM cells from AR^tfm^ mice plated on methylcellulose showed fewer macrophage and total granulocyte (G) and macrophage (M) colonies in male HFD AR^tfm^ animals (Fig. 7*J*) than WT males. Overall, these results emphasize a role for androgens in driving weight gain with HFD, myeloid expansion, metabolic inflammation, and glucose tolerance.

## Discussion

Sex hormones play a role in the onset of metabolic disorders, but their role in the development of these disorders remains unclear (36). Although studies have evaluated metabolic changes in models of obesity using both male and female animals, there is a paucity of studies that directly compare loss of gonadal hormones in male and female animals. In our study, we directly compare both gonadectomized males and females and investigate specific hormone receptor–mediated mechanisms of metabolic and myeloid inflammatory responses to HFD. We hypothesized a proinflammatory role for androgens in obesity and a protective anti-inflammatory role of estrogens, given the observation that metabolic inflammation is increased in male animals. We did find that GX enhanced adipose tissue inflammation in obese females but with only moderate changes in metabolism. Surprisingly, while GX male animals on HFD had improved metabolic outcomes, these were not associated with improvements in adipose tissue inflammation and were associated with increased CD11c^+^ ATMs and increased proinflammatory cytokines. On the other hand, circulating Ly6c^hi^ monocytes and BM myeloid potential was dampened with CAS, suggesting different effects of androgens during leukocyte development *versus* polarization in tissues.

Given the complexity of peripheral hormone metabolism, we investigated the direct role of hematopoietic ERα and found almost no effect on metabolism or tissue inflammation. AR deficiency however improved metabolic and inflammatory responses to HFD, suggesting that loss of AR signaling beginning during development, with intact production and metabolism of androgen, leads to both improved metabolism and decreased ATMs. Overall, these results emphasize that androgens play significant roles in immune responses and metabolic responses, with direct roles in activating BM immune cells and monocytes, and in regulating inflammatory tone in the adipose tissue (Table 1). These studies also emphasize that there can be a disconnect between adipose tissue inflammation and metabolism as has been seen in studies of weight loss (37).

**Table 1:** Summary of key findings from each experimental group

Experimental model	Adiposity	Glucose tolerance	Adipose CD11c^+^ ATMs	BM myeloid CFU
OVX *versus* sham HFD	Increased	Mild impairment	Increased	Increased
Hematopoietic ER⍺^−/−^*versus* WT	Unchanged	Unchanged	Unchanged	Increased in ND
CAS *versus* sham HFD	Increased	Improved	Mild increase	Decreased
AR^tfm^*versus* WT HFD	Unchanged	Improved	Decreased	Decreased

Our data demonstrate that male GX improves glucose tolerance and insulin sensitivity with no improvement in adipose tissue inflammation. Lifelong AR deficiency improves both metabolic and inflammatory responses to HFD in male mice. *In vitro*, male GX and AR^tfm^ mice have dampened myeloid colony production, and consistent with this, the addition of testosterone enhances myeloid colony production. These discordant results of adipose inflammation in the two different models of androgen signaling deficiency could be due to the developmental timing of androgen signaling or result from altered circulating hormone levels that result from receptor deficiency with retained androgen production. The later possibility is supported by the finding that isolated BM either from HFD-exposed males or fatty acid exposure with testosterone *in vitro* had increased myeloid expansion. Prior work consistently demonstrates that GX or AR deficiency exacerbates adiposity and adipocyte hypertrophy, but the effects on glucose tolerance (32, 33, 38) and adipose inflammation have been mixed. These differences in findings may be due to methodology, including fat content in diet, doses used in glucose challenges, and even length of diet challenge. It is known that AR is important for insulin production (21, 39), and the degree of hyperglycemia in high GTTs may outstrip the capacity for insulin production regardless of insulin sensitivity.

While androgens may alter metabolic responses to obesity (32, 33, 38), the contribution of androgen signaling to the myeloid expansion in HFD has not been examined (19, 40). Our *in vitro* studies with testosterone stimulation demonstrated the inflammatory role of androgens by enhancing myelopoiesis in WT males, which was dampened in BM from AR^tfm^ and GX males. Our androgen deficiency and male GX models also showed decreased monocytes. However, GX and AR^tfm^ showed discordant results in abundance of ATMs. Furthermore, we found increased levels of proinflammatory cytokines in the adipose tissue after CAS with HFD. These cytokines may be directly expressed by the enlarged adipocyte mass (41). Interestingly, our finding of similar ATMs was seen in BM chimeras with AR^−/y^ BM (38); however, those animals had decreased liver macrophages. Taken together, these findings further emphasize that there may be differences in inflammatory roles for androgens in the BM, monocytes, and tissues. A major limitation of our studies is that these models are whole body knockouts of androgen signaling. Therefore, differences in tissue-specific cues for trafficking and accumulation of immune cells are not controlled, and further, our findings on metabolism may be a result of whole-body effects and not solely on due to adipose-specific inflammatory responses.

Rodent model studies of estrogen deficiencies as well as clinical studies in women with polycystic ovarian syndrome, postmenopausal women, and aromatase-deficient men (42, 43) demonstrate protective effects of estrogen on metabolic function by reducing proinflammatory cytokines and maintaining insulin sensitivity during obesity (12, 20, 44, 45). Our studies utilizing OVX demonstrated modest worsening of glucose tolerance, impaired energy expenditure, increased adipose tissue inflammation, and enhanced myeloid proliferation in the BM in the context of HFD, supporting a moderate protective role of estrogen compared with the stronger deleterious effect of testosterone.

Prior studies suggest that ERα may be the primary mediator of estrogen signaling in both adipose tissue and the immune system (26). However, deletion of ERα from the BM did not recapitulate either the immune effects or the metabolic effects of OVX. This may be due to estrogen activity on peripheral cell types driving inflammation or other ERs (G protein–coupled estrogen receptor or ERβ) that may play an important role. Studies examining adipose tissue ERα found protective effects of estrogen signaling on adiposity and metabolism, however, flow cytometric characterization of ATMs was not performed in these studies (46). Furthermore, the study that suggested a significant immune role for BM ERα was performed on a leptin-deficient background, which may significantly alter immune responses compared with HFD alone (26).

Another limitation of our study is that we only assessed circulating serum hormone levels, whereas other sources were not determined. The endocrine model overlooks the extensive capacity of adipocyte cell types including immune cells to generate and metabolize sex steroids, enabling the production of sex steroids for autocrine and intracrine signaling (47, 48) that may possibly explain some marginal effects on inflammation as in BM ERα knockout experiments. Sex differences in metainflammation have also been seen in other metabolic tissues during obesity (49), including the liver (50) and myocardium (51). Our study is limited as we focused on the adipose tissue, and future studies will need to study the role of sexual dimorphism in inflammatory responses in other metabolic organs.

In summary, females remain protected from HFD-induced reprogramming of HSCs, ATM accumulation, and insulin resistance, similar to premenopausal women with obesity (52, 53). Our studies provide evidence that androgen deficiency even with HFD improves metabolism, enhances adiposity, and improves BM and peripheral myeloid inflammation, as demonstrated in our GX and AR^tfm^ studies. The AR^tfm^ studies add that the removal of AR receptor alone dampens both inflammation and metabolic responses to HFD. Further studies examining inflammation with cell-specific knockout of AR and ERs will be critical to decipher the contribution of peripheral tissues and specific immune cells to inflammatory responses in obesity.

## Experimental procedures

### Animal studies

C57Bl/6J (000664), AR testicular feminization AR^tfm^ (001809) mice, which have a spontaneous mutation in the AR gene (54, 55), and ERα knockout male and female (004744) mice were purchased from Jackson Laboratories. Appropriate background control mice and littermate controls from Jackson were used for genetic knockout models. Gonadectomized and sham surgery animals were purchased from Jackson Laboratories at 4 weeks of age. All mice were fed *ad libitum* either a control or an ND consisting of 13.5% fat (5LOD; LabDiet) or HFD consisting of 60% of calories from fat (Research Diets; D12492), starting at 6 weeks of age for 16 weeks of duration. Animals were housed in a specific pathogen-free facility with a 12-h light/12-h dark cycle and given free access to food and water. Animal protocols were in compliance with the Institute of Laboratory Animal Research Guide for the Care and Use of Laboratory Animals and approved by the University Committee on Use and Care of Animals at the University of Michigan (animal welfare assurance number: A3114-01). Metabolic cage studies were performed in singly housed animals in the Michigan Mouse Metabolic Phenotyping Center with evaluations for 3 days (13).

### Metabolic studies

Metabolic testing included fasting glucose and serum collection after 10 weeks of HFD, intraperitoneal GTT at 12 weeks of HFD, and intraperitoneal ITTs at 14 weeks of HFD. GTTs were performed with 0.7 g/kg of d-glucose and ITTs with 1 unit/kg of humulin both after 6 h of fasting.

### BM transplantation studies

Bone marrow transplantation (BMT) studies were performed as previously described (19). BM from donor groups (10 million cells/mouse) was injected retroorbitally into lethally irradiated (900 rad) recipient mice (6 weeks of age). Animals were treated with antibiotics in water (polymyxin and neomycin) for 4 weeks post-BMT. Donors for these experiments were males and females either WT or ERα^−/−^ mice. Recipients were sex-matched WT animals. Half of the animals per group were started on HFD 6 weeks after BMT.

### Adipose tissue stromal vascular fraction isolation and flow cytometry

Adipose tissue fractionation and flow cytometry analysis were performed as described previously (19). Briefly, whole adipose tissue was minced and digested with type II collagenase (Sigma; 1 mg/ml in RPMI media) for 15 to 30 min at 37 °C on a rocker. Filtrated samples were spun at 500*g* for 10 min, and red blood cell lysis was conducted (Biosciences; 00-4333-57). Stromal vascular fraction cells were stained with antimouse CD45 eFluor450 (30-F11 monoclonal; Invitrogen), CD64 PE (X54-5/7.1 monoclonal; BD Pharmingen), and CD11c APC or eFluor 780 (N418 monoclonal; Invitrogen), and gating was performed for macrophage populations and by CD45 gates to determine ATMs (13). T-cell stains included CD3 PerCP5.5 (145-2C11), CD4 APC (GK1.5), CD8 FITC (53-6.7), and FoxP3 PE (NRRF-30). All hematopoietic stem and progenitor cell stainings were performed using lineage staining on APC including CD4 (GK1.5), CD5 (53-7.3), CD8 (53-6.7), CD11b (M1/70), B220 (CD45R) (RA3-6B2), Gr1(RB6-8C5), and Ter119 (eBioscience), Sca PECy7 (D7), CD117 APCCy7 (2B8), Endoglin/CD105 PacBlue (MJ7/18), CD16/32 PerCP5.5 (93), CD150 PE (TC15-12F12.2), and CD48 FITC (MEM-102) as previously described (17).

### Immunofluorescence and immunohistochemistry

Adipose tissue was fixed in 1% paraformaldehyde for 24 h and then transferred to PBS at 4 °C. Tissues were blocked in 0.3% Triton, 5% bovine serum albumin, and then stained using polyclonal anti-caveolin (CAV-1; Cell Signaling) and anti-Mac2 (MAC-2; Galectin-3; eBioM3/38). For histology, tissues were formalin fixed, paraffin embedded, sectioned at 5 μm, and stained with H&E. H&E staining was performed by the University of Michigan's Comprehensive Cancer Center Histology Core. Images were captured with an Olympus IX-81 fluorescent microscope. Adipocyte sizing was conducted by capturing multiple TIFF-gray-scale images, and adipocyte circumference was determined with ImageJ software (the National Institutes of Health). Pixel areas of all individual cells in 3 to 5 areas were analyzed and averaged for each condition.

### Methylcellulose CFU assay

BMs from femurs were flushed with Iscove's modified Dulbecco's medium, treated with fatty acid–free bovine serum albumin or 10 μM palmitic acid for 1 h at 37 °C, and then resuspended in MethoCult (Stem Cell Technologies) medium and plated (10,000 cells/plate) for granulocyte (G)/macrophage (M) assay. Testosterone (Sigma; T1500) was added at a concentration of 10 μM. CFUs were counted 7 days after plating. CFUs were further classified into granulocytes (G) and macrophages (M) based on size of the colonies (https://cdn.stemcell.com/media/files/manual/MA28405-Mouse_Colony_Forming_Unit_Assays_Using_MethoCult.pdf).

### Quantitative real-time PCR

RNA was extracted from adipose tissue using Trizol LS (Life Technologies), and complementary DNA was generated using a High-Capacity cDNA Reverse Transcription Kit (Applied Biosystems). SYBR Green PCR Master Mix and the StepOnePlus System (Applied Biosystems) were used for real-time quantitative PCR. *GAPDH* expression was used as an internal control for data normalization. Samples were assayed in duplicate, and relative expression was determined using the 2^−ΔΔ^CT method. All primers used are listed in the supporting table.

### Hormone and TG measurements

Estradiol and testosterone levels were assessed with Cayman ELISA kits (estradiol, catalog no.: 582251 and testosterone, catalog no.: 582701). Serum TG was measured with Infinity TG determination kit (Thermo Fisher Scientific), following manufacturer's instructions. Liver TGs were homogenized, extracted by a modified Folch method, and assayed (18).

### Statistical analyses

The data from each experiment were analyzed using univariate methods, and when appropriate, multivariate methods were used with profile analysis or repeated-measurement analysis. All results adjust for multiple comparisons. Univariate analysis was carried out using general linear models to identify the significant factors, and Tukey's pairwise comparisons were used to identify the pairs of groups that were significantly different. For variables, such as GTT and ITT, where measurements are taken at different time points, multivariate ANOVA was used to identify the significance of the factors and their interactions. The programs used for the analysis were MINITAB (Minitab, LLC) and JMP (SAS Institute).

## Data availability

The datasets generated during and/or analyzed during the current study are available from the corresponding author upon reasonable request.

## Supporting information

This article contains supporting information.

## Conflict of interest

The authors declare that they have no conflicts of interest with the contents of this article.
