# Supplementary material for: Sex hormones regulate metainflammation in diet-induced obesity in mice
Source: J Biol Chem. 2021 Sep 29;297(5):101229. doi: 10.1016/j.jbc.2021.101229 (PMC8526779; doi:10.1016/j.jbc.2021.101229)
Supplement: Supplemental Figure S2 [file mmc2.pdf]

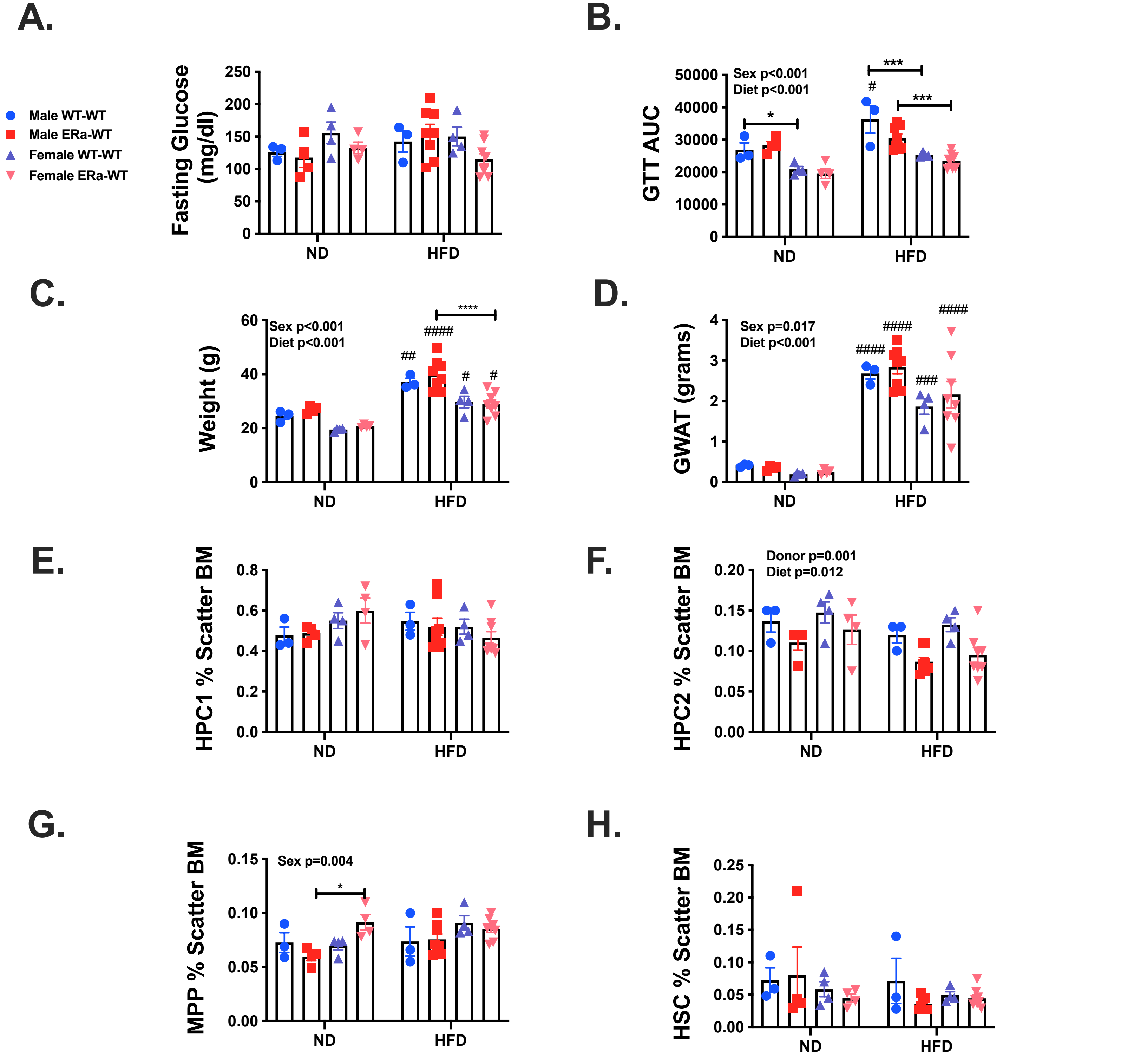

Supplementary Figure 2: Hematopoietic ER$^{-/-}$ metabolic parameters and progenitor populations. Male and female bone marrow transplants (BMT) were generated with either ER$^{-/-}$ or WT BM. Animals were placed on either normal diet (ND) or high fat diet (HFD). Donor and recipient sex were the same in these studies. 10 week fasting glucose (A) was not significantly different amongst groups. GTT area under the curve (B) did demonstrate differences by sex and diet but not different based on ER$^{-/-}$. After 16 weeks, weight (C) and GWAT weight (D) were increased by diet in males more than females equally by genotype. Bone marrow was assessed for hematopoietic progenitor cell population 1 (HPC1) (E), HPC2 (F), multipotent progenitors (MPP) (G) and (Hematopoietic stem cells (HSCs) (H) and these were not different by genotype. N=4-8 per group. Data shown as average +/- SEM. *p<0.05, **p<0.01, ***p<0.005, and ****p<0.001. #p<0.05, ##p<0.01, ###p<0.005, and ####p<0.001 marks differences between ND and HFD of the same sex and donor group.
