# Supplementary material for: Sex hormones regulate metainflammation in diet-induced obesity in mice
Source: J Biol Chem. 2021 Sep 29;297(5):101229. doi: 10.1016/j.jbc.2021.101229 (PMC8526779; doi:10.1016/j.jbc.2021.101229)
Supplement: Supplemental Table S1 [file mmc3.docx]

|  | **Forward Primer** | **Reverse Primer** |
| --- | --- | --- |
| *Gapdh* | TGAAGCAGGCATCTGAGGG | CGAAGGTGGAAGAGTGGGAG |
| *Ir* | TTTGTCATGGATGGAGGCTA | CCTCATCTTGGGGTTGAACT |
| *Irs1* | CGATGGCTTCTCAGACGTG | CAGCCCGCTTGTTGATGTTG |
| *Il4* | GGTCTCAACCCCCAGCTAGT | GCCGATGATCTCTCTCAAGTGAT |
| *Il6* | TAGTCCTTCCTACCCCAATTTCC | AAGGAACCCTTAGAGTGCTTACT |
| *Mcp1* | TTAAAAACCTGGATCGGAACCAA | GCATTAGCTTCAGATTTACGGGT |

**Table 1. Primer sequences**

|  |
| --- |
| \|  \| \| --- \| \|  \| |
| \| \|  \| \| --- \| \|  \| \| \| --- \| --- \| --- \| \|  \| |
|  |
