# Supplementary material for: Sex hormones regulate metainflammation in diet-induced obesity in mice
Source: J Biol Chem. 2021 Sep 29;297(5):101229. doi: 10.1016/j.jbc.2021.101229 (PMC8526779; doi:10.1016/j.jbc.2021.101229)
Supplement: Supplemental Figure S1 [file mmc1.pdf]

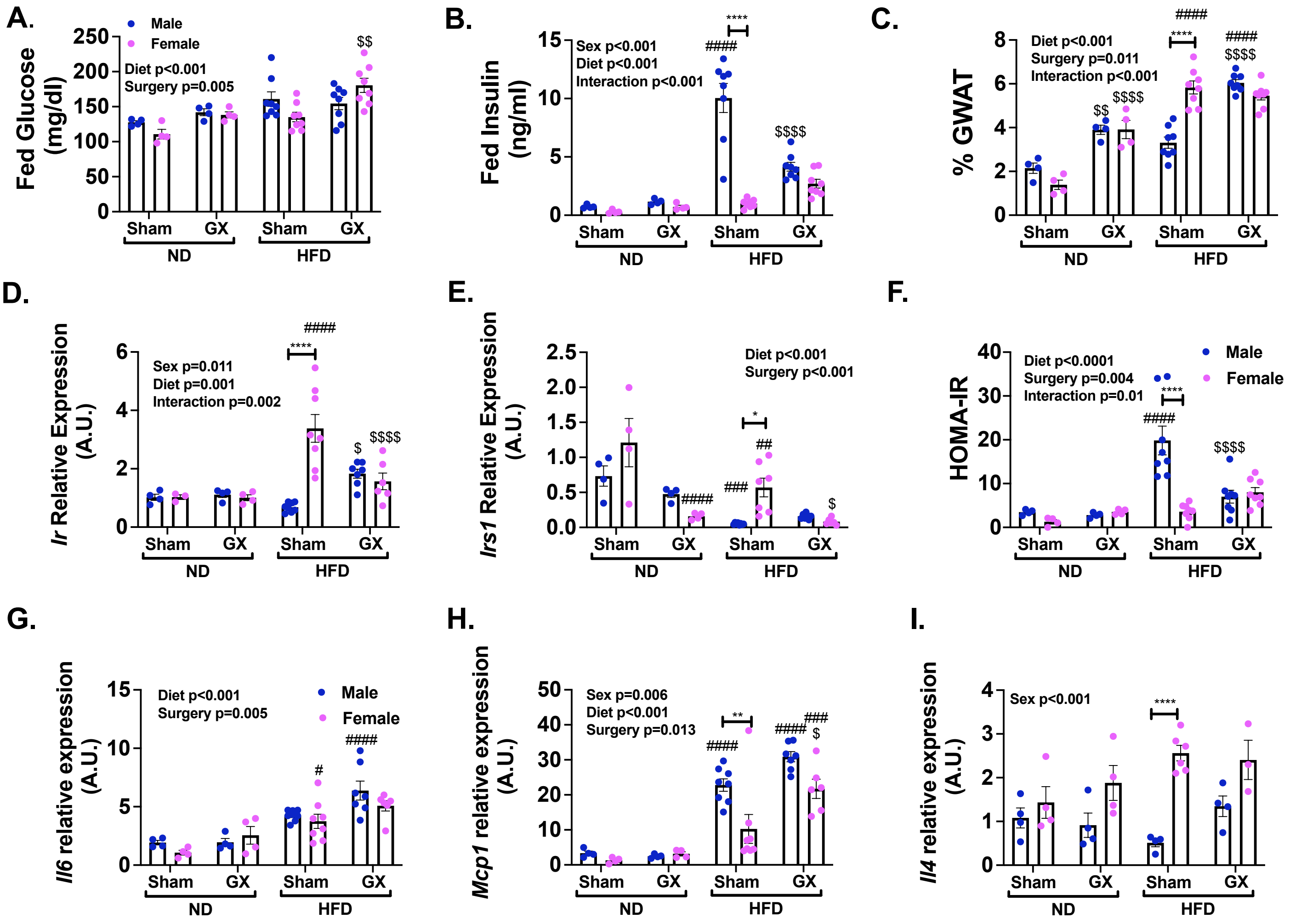

Supplementary Figure 1: Fed metabolic parameters and gene expression in sham and gonadectomy GWAT. Male and female animals underwent sham surgery or gonadectomy (GX) at 4 weeks of age and then at 6 weeks of age remained on ND or started HFD. Fed glucose (A) varied significantly by diet and surgery but was highest in female GX HFD mice. Fed insulin (B) was markedly higher in male sham HFD animals and attenuated by GX. % GWAT at 16 weeks (C) varied significantly by diet and surgery and was higher in female sham HFD animals with attenuated differences by sex after GX. Gene expression of visceral fat (GWAT) was conducted and normalized to *Gapdh. Ir* (D) and *Irs1*(E) gene expression were lowest in Male sham HFD animals and (F) calculated HOMA-IR was highest in sham HFD males. *Il6* and *Mcp1* (G and H), were highest in GX HFD animals of both sexes, while *Il4* (I) was increased in females relative to males in all conditions. N=4 for ND groups and N=6-8 for HFD groups. Data shown as average +/- SEM. *p<0.05, **p<0.01, ***p<0.005, and ****p<0.001 between male and female of each group. #p<0.05, ##p<0.01, ###p<0.005, and ####p<0.001 marks differences between ND and HFD of the same sex and surgery group. $ p<0.05, $$p<0.01, $$$p<0.005, and $$$$p<0.001 marks significant differences by surgery.
